# COVID-19 poses novel challenges for global primary care

**DOI:** 10.1038/s41533-020-0187-x

**Published:** 2020-06-18

**Authors:** Siân Williams, Ioanna Tsiligianni

**Affiliations:** 1 International Primary Care Respiratory Group (IPCRG), London, UK; 2 Health Planning Unit, Department of Social Medicine, University of Crete, Heraklion, Greece

## Abstract

COVID-19 is presenting novel challenges for global primary care, and its response has been inspiring, varied, and also troubling.

Let us start with the good news.

There has never been more interest in breathlessness: how it feels, how to diagnose it, how to treat it, and how to assess and support recovery. This presents opportunities for the primary care respiratory community to influence and encourage research priorities, and highlights the need to share knowledge through scholarly publication. For example, the breathlessness algorithm and its development process by the Primary Care Respiratory Society and the British Thoracic Society was not widely followed, because breathlessness did not fit the silo-planning of many health services. Instead, it was built on clinical experience and knowledge categorised by symptom not disease (www.respiratoryfutures.org.uk/resources/impress-documents/impress-breathlessness-algorithm/). Now, with COVID-19, there is a new urgency. We have seen examples of assessment algorithms^1^ recommending new tools that almost immediately are rejected as unworkable, such as the Roth test (www.cebm.net/). We encourage primary care respiratory-interested clinicians and academics to seize the moment to propose, test and publish tools to assess breathlessness in a primary care consultation and also online where usual questions may no longer be helpful.

## Public health conversations

There is a public realisation that public health and individual health are entangled, and that “health in all policies” is genuinely relevant and important. Our individual health is entangled with those we live and work with, with people on the other side of the world and with our environment (www.cognitive-edge.com/blog/connections-not-things/).

The public has become aware of different health systems in other parts of the world, including those in low- and middle-income countries, and recognise the strengths and weaknesses of their own, and that decisions about public health are part of a political process that they can influence through their vote. The role of different stakeholders, such as religious leaders, in health is also being recognised (www.afro.who.int/news/religious-leaders-join-covid-19-fight-africa).

## Vaccines and vaccination programmes

There is a renewed conversation about the importance of vaccines, which may help buck the worrying trend in vaccine hesitancy and the need to earn public trust in healthcare worker advice, identified by the WHO in January as one of the biggest challenges we face in the next decade (presciently, together with preparing for epidemics and investing in the people who defend our health) (www.weforum.org/agenda/2020/02/who-healthcare-challenges-2020s-climate-conflict-epidemics). This includes not only the hope of a vaccine for COVID-19, but encouraging countries to protect existing immunisation programmes so that momentum is maintained in controlling other vaccine-preventable diseases.

## Global collaboration

Connections within the IPCRG network across low-, middle- and high-income countries are more active than ever, building on shared language. For example, Spanish and Portuguese General Practitioners (GPs) and community pharmacists are freely sharing their infection control experience and resources with peers in Latin America so that they learn from successes and failures. IPCRG webinars are planned for GP and pharmacist colleagues in English-, French- and Portuguese-speaking Africa.

## Asthma and chronic obstructive pulmonary disease (COPD) management

There is increasing public interest in asthma and COPD treatments. As people who have not been on the primary care “radar” seek safe levels of supply of their inhalers, there are opportunities to teach and enhance understanding. It is a moment to ask, advise and act on inhaler technique and the importance of inhaled corticosteroids in asthma. In Malaysia, the Thoracic Society and local IPCRG group acted on home and community-based nebulisation for asthma. As an aerosol-generating procedure, nebulisation could present unnecessary risks compared to guideline-advised inhalers and large-volume spacers. However, when spacers are not on the national formulary, this presents a significant out-of-pocket expense requiring policy changes to ensure access. In Australia, following the bushfires and now COVID-19, problems of low stock caused by the easy availability of over-the-counter short-acting beta-agonists for asthma gained policy attention. We urge regulators to support “right care” by restricting this access and improving access to inhaled corticosteroids.

It is now widely accepted that asthma and COPD are ambulatory care-sensitive conditions, and that good primary care management, in particular one that offers continuity of care and addresses multi-morbidity, can avoid unplanned hospital admissions^2^. A recent rapid review on 22 April 2020 of hospitalisation data shows that this can also be important during this pandemic by keeping beds free for people with COVID-19 (www.cebm.net/oxford-covid-19-evidence-service/). We urge colleagues from around the world to continue to publish their findings about the most effective interventions for managing asthma and COPD in primary care, including how to maintain continuity of care despite pressures of a pandemic.

Observational data suggest that things are going reasonably well. People with asthma and COPD are not over-represented in hospitals, although if they do catch COVID-19, people with COPD, and possibly asthma, are at greater than average risk of more severe problems^3^. Let us collect and share the data, and more importantly, explore the reasons behind the lower than expected healthcare utilisation. Is it a function of good-quality primary care taking advantage of “teachable moments”, better adherence to prescriptions for effective medicines, better hand hygiene leading to fewer COPD exacerbations, self-isolation leading to less exposure to intergenerational contact and outdoor asthma triggers such as pollen and/or lower air pollution? Or are people delaying seeking care, potentially worsening outcomes?

## Tobacco dependency

COVID-19 presents a powerful reason to quit tobacco. We applaud the WHO’s strong stance. Whilst we await evidence, there is concern that waterpipe and cigarette smoking increase the risk of infection^4^. People with a history of tobacco use are also more at risk of respiratory infections and may get worse symptoms if they get COVID-19. Therefore, it is a good time for primary care to support smokers who want to quit, because the evidence is strong about the increased impact of a supported quit attempt^5^. We urge primary care pharmacists, GPs and other healthcare workers to actively seek out and support their tobacco-dependent populations to quit. Hypothetical benefits of nicotine in acute COVID-19 does not alter the public health message of the danger of smoking (www.qeios.com/read/FXGQSB).

Now let’s move to the less favourable news.

## Protecting primary care

We must ensure that despite the current and appropriate focus on hospitals, advocacy for the role of primary care in achieving health equity through universal health coverage (UHC) is maintained and reinforced. Furthermore, we must advocate for respiratory services as part of UHC. This means continuing to strongly support and invest in the World Health Organization (WHO), the champion of UHC (www.bjgplife.com/2020/04/20/untitled/).

The IPCRG network must advocate for national investment in the recruitment, teaching and protection of primary care and access to affordable inhaled medicines and spacers, tobacco dependence treatment and rehabilitation. For example, whilst nicotine replacement therapy (NRT) is on the WHO essential medicines list, it is apparently on national formularies in only 17 countries in the world, revealing a significant unmet need that should be addressed whatever the findings of COVID-19 studies (www.global.essentialmeds.org/dashboard/medicines/1306).

We also know that well-organised primary care, comprising expert generalists rooted in their local neighbourhoods, is well-placed to manage multi-morbidity^6^. This role remains crucial to avoid excess morbidity and mortality due to interruption of routine long-term condition care. In addition, older people with multi-morbidity are most at risk of COVID-19, and primary care must continue to provide safe, compassionate and effective ways to protect and care for them^7^. This means that during periods of physical distancing and protection of vulnerable people through self-isolation, primary care remote consultations are needed. These can be effective, efficient and enable the responsive involvement of secondary care colleagues. In Brazil, we have seen the value of remote consultations, to enable primary care to be supported to deliver good-quality asthma care^8^. This remains a good way forward during COVID-19, and a small mark of success should be direct contact between hospital respiratory teams and GPs.

## Technology: equitable access to care, health information and privacy

We are seeing the widespread use of remote patient consultations by phone, email or video to protect both healthcare professionals and patients, even in highly stressful situations of loved ones dying or bereavement. Some remote consultation may persist, and improve acceptability, efficiency and cost of health service delivery as we follow the “desire lines” of the public in how they access primary care. However, we need to target care to ensure health equity is addressed and improved. For example, remote consultations for mild uncomplicated conditions might release face-to-face resource for those with more severe or complex problems. Health information must be provided for the estimated 40% of the world not yet online. We need to explore how to ensure no one is left behind and actively seek to address social exclusion of the poorest and women, whose access to education still lags behind men (https://bit.ly/3cJonfE). We also need to monitor carefully the impact of a reduction in face-to-face care on consultations for time-critical conditions such as early diagnosis for cancer and pain relief.

IPCRG is a signatory to an open letter to the UN Secretary General asking for coordination of a global equity-focused response to COVID-19 by setting up a multi-sector “Global Health Equity Task Force” to confront the impact of the pandemic in all its dimensions. Sign up to support www.sustainablehealthequity.org/.

Privacy fears will also need to be addressed as governments and major technology firms introduce tools for contact tracing and rules governing patient (and carer) access to care records evolve^9^.

## Care for frail and older people

At last there is a conversation between politicians, health policy experts and the public about the challenges of providing compassionate and safe care for our frailer citizens, knowing that up to 50% of deaths have occurred in care homes (www.bit.ly/2XbQayR). Some countries, such as Greece, with far less reliance on nursing home care, and more home-based care, have protected their frail citizens much better (www.bit.ly/3bLerki). Primary care should take the opportunity to join and then steer the conversation to new sustainable and safe models of care.

## The education challenge

COVID-19 presents many of us with decision-making challenges due to the level of uncertainty. Whilst politicians take responsibility for making decisions at the national level, primary care has to take responsibility for helping individual patients make decisions. This requires high levels of competence in risk communication in the absence of little—and often changing—evidence. Over time, knowledge about issues that concern global IPCRG members such as testing for COVID-19, the value of masks for stopping spread, the effectiveness of nasal saline irrigation, how to differentiate between an asthma attack and COVID-19 respiratory problems, the value of inhaled corticosteroids in COVID-19, the impact of quitting tobacco and the health sequelae of both COVID-19 and the long-term home isolation for older people will become clearer. However, for now, we need to share experience and work with behavioural experts to optimise communication of personal risk and co-create effective behaviours. We anticipate a continued need both for good-quality evidence and also education programmes about how to teach patients and the public about risk of a significant hazard when there is so much uncertainty.

## Mental health

Anxiety, depression and trauma linked to social isolation, financial and employment loss, bereavement, alcohol overuse and tobacco consumption, risk of domestic violence (www.bit.ly/2X6pijv) and COVID-19 itself require primary care to hold on to its goal of parity of esteem between physical and mental health and find ways to identify, counsel and support patients in the months ahead. In addition, we need to offer appropriate mental health support to clinicians who have also been affected by trauma and, in fee-for-service payment systems, potentially financial loss given the reduction in consultation rates. We seek evidence about how best to offer care but also advice. We anticipate that there will be a need for primary care to play its public health role in mitigating the effects of potential economic recession by identifying at-risk populations early, discussing finances, and offering information about any potential support. This is likely to require training and also draw on primary care’s deep understanding of its neighbourhood^10^.

## End-of-life and bereavement support

Finally, COVID-19 can be a lethal infection, people are dying, and will continue to die even in countries with good access to intensive care facilities. Colleagues are already providing compassionate end-of-life care and bereavement support in new ways for those prevented from physical contact and the usual acts of kindness and grieving rituals^11^. We must share resources to improve capacity to deliver advanced breathlessness support and good end-of-life conversations (www.bit.ly/2LD7FCD). New ways need to be found in a COVID-19 world (www.nice.org.uk/guidance/ng163/chapter/6-Managing-breathlessness).

## Concluding remarks

Whilst there remains urgent and vitally important questions about equal access for all to care for physical and mental health, and how to keep care workers safe, there is plenty of room for hope. Let us together advocate for the role of primary care at an individual, local, national and international level in managing COVID-19 and its long-term impact as well as routine respiratory care, and for investment in capacity building to ensure that when universal access is achieved, it is to the right, good-quality care^12,13^. Let us also produce good-quality evidence to underpin this.
